# Ventromedial Nucleus of the Hypothalamus Neurons Under the Magnifying Glass

**DOI:** 10.1210/endocr/bqab141

**Published:** 2021-07-15

**Authors:** Tansi Khodai, Simon M Luckman

**Affiliations:** Faculty of Biology, Medicine and Health, The University of Manchester, Oxford Road, Manchester, UK

**Keywords:** VMH, body weight, glucose, counter-regulatory response, sexual dimorphism

## Abstract

The ventromedial nucleus of the hypothalamus (VMH) is a complex brain structure that is integral to many neuroendocrine functions, including glucose regulation, thermogenesis, and appetitive, social, and sexual behaviors. As such, it is of little surprise that the nucleus is under intensive investigation to decipher the mechanisms which underlie these diverse roles. Developments in genetic and investigative tools, for example the targeting of steroidogenic factor-1-expressing neurons, have allowed us to take a closer look at the VMH, its connections, and how it affects competing behaviors. In the current review, we aim to integrate recent findings into the literature and contemplate the conclusions that can be drawn.

As basic and translational scientists, we invest immense effort into understanding the vital physiological and behavioral patterns of mammals. This is because the optimal regulation of key parameters, such as glucose and energy, are vital for good health. Increasing our knowledge is most pertinent today, as modern lifestyles are contributing to several disorders associated with the imbalance of homeostasis. For example, the worldwide prevalence of diabetes has doubled in the last 20 years and continues to rise. Currently, the only effective treatment for late-stage diabetes is insulin treatment, so there is an urgent need to understand how our brain modifies physiology in order to identify alternative therapeutic avenues. Our behavior is of paramount importance to the maintenance of balance in individuals and in societies. Specific behavioral adaptations, such as modulating our food intake, are imperative for glucose and body weight homeostasis, whereas by controlling aggression, we can reduce harm caused to ourselves and to others.

The brain circuits involved in the control of such diverse human traits are complex and often interlinked; making it extremely difficult to study them. Adding to the complexity are the sex differences observed in physiology (1) and behavior (2). The ventromedial nucleus of the hypothalamus (VMH) is 1 of the few brain structures that is anatomically preserved across mammalian species, and is involved in the regulation of many different aspects of both physiology (3-6) and behavior (7).

Seminal work (mostly on rodents) revealed that, while the majority of VMH neurons are glutamatergic (8), the nucleus accommodates a heterogeneous mixture of cell types (Fig. 1 and Table 1), differentiated by their expression of markers such as pituitary adenylate cyclase-activating peptide (PACAP) (18), nitric oxide synthase 1 (NOS1) (19), brain-derived neurotrophic factor (BDNF) (20), estrogen receptor alpha (ERα) (21) and leptin receptor (LepR) (22). However, it is the expression of the steroidogenic factor-1 (SF1) that is often seen as an exclusive marker for the VMH. SF1 (gene name *Nr5a1*) is a transcriptional regulator, crucial for the correct development of the adrenal gland, gonads, pituitary gonadotropes, and, within the brain, the VMH (23). The exclusivity of SF1-positive neurons to the VMH has provided an opportunity to explore this nucleus more closely. Two recent, single-cell sequencing studies have attempted to further elucidate the phenotype of VMH cells. Kim and co-workers (24) used an unbiased approach and sampled the VMH anatomically from adult mice. They found that *Nr5a1* is expressed mostly in the anterior, central, and dorsomedial VMH (dmVMH), confirming an earlier observation that neurons in the adult ventrolateral VMH (vlVMH) do not express SF1 (25). There were no obvious distinguishing markers for neurons in the anterior or central VMH, whereas 17 transcriptomic clusters were identified in the vlVMH (24). By contrast, van Veen et al. (26) sorted VMH cells from a *Nr5a1*-Cre × fluorescent reporter mouse line, thus sampling from all neurons expressing SF1 developmentally. They identified only 6 transcriptomic clusters, which they classified according to the topmost differentially expressed (though not exclusive) transcript in each cluster: *Tac1*, *Rprm*, *Pdyn*, *Sst*, *Hpcal1*, or *Gal* (Table 2). The importance of these different clusters and how they relate to more classically defined populations needs to be elucidated.

**Table 1.** Summary of the distribution of VMH neuronal markers across different divisions of the VMH in adult mouse brain

Neuronal marker	Anatomical division of VMH (adult mouse brain)			Techniques	Reference
	Dorsomedial	Central	Ventrolateral		
BDNF	++	++	++	ISH	(9)
CCK_A_	—	—	++ (female)	ISH	(10)
CCK_B_	++	++	+	*Cckbr*-Cre × Rosa26^eGFP^	(11)
ER⍺	+	—	+++	ISH, autoradiography	(12)
ERβ	—	—	++	ISH, autoradiography	(12)
LepRb		++		ISH, *Leprb*-i-Cre x Rosa26^eYFP^	(13)
NOS1	+	—	++	IHC	(14)
PR	—	—	++	Gene targeted expression of placental alkaline phosphatase (PLAP)	(6)
PACAP	+	++	++	ISH, *Pacap*-i-Cre x Rosa26^eYFP^	(15,16)
SF1	+++	++	+	ISH, IHC	(17)

Relative density: +++ high, ++ moderate, + low.

ISH, in situ hybridization histology; IHC, immunohistochemistry.

**Table 2.** RNA sequencing of fluorescence-sorted VMH cells from a *Nr5a1*-Cre × fluorescent reporter mouse line were attributed to 6 clusters by van Veen et al. (26)

Gene	Name	General Function	Reference
*Tac1*	Tachykinin precursor 1	Encodes peptides belonging to the tachykinin family: substance P, neurokinin A, neuropeptide K and neuropeptide gamma	(27)
*Rprm*	Reprimo	Possibly involved in p53 mediated G2 arrest of cell cycle	(28)
*Pdyn*	Prodynorphin	Encodes precursors of the opioid peptides: beta-neoendorphin, dynorphin, leu-enkephalin, rimorphin and leumorphin	(29)
*Sst*	Somatostatin	Encodes somatostatin precursor which is an important regulator of the endocrine system, with an inhibitory role on the release of growth hormone and thyroid-stimulating hormone	(30,31)
*Hpcal1*	Hippocalcin-like 1	Encodes a neuron-specific calcium-binding protein found predominantly in the retina and brain	(32)
*Gal*	Galanin and GMAP prepropeptide	Encodes neuroendocrine peptides, including galanin, that are involved in several neurological functions such as feeding and energy balance	(33)

Van Veen et al. assigned each a description based on the most abundant transcript, though these transcripts are not necessarily exclusive to any particular cluster. An annotation of each of these 6 assigned genes is provided

**Figure 1.**
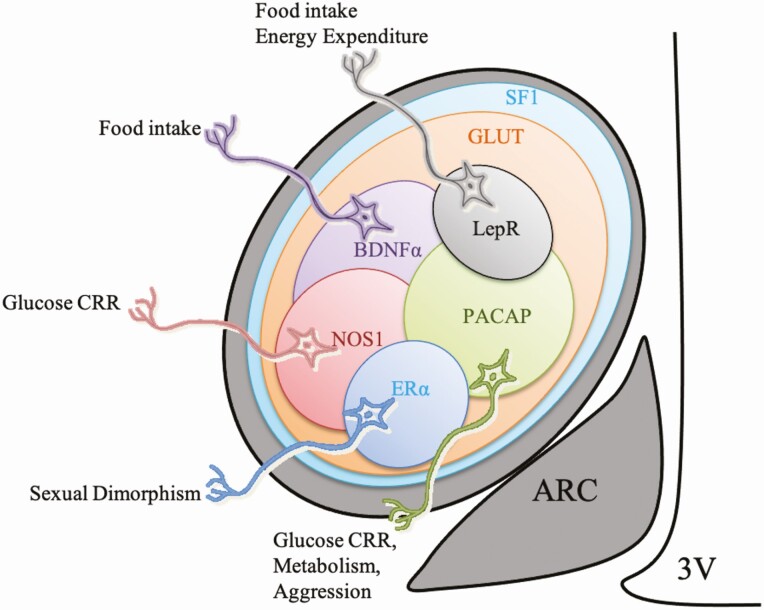
Schematic representation of the identified neuronal populations within the VMH involved in modulation of different homeostatic and behavioral responses.

In this review, we summarize how recent studies have built on our knowledge, and suggest care is needed to allow accurate interpretation of observations made following VMH neuron manipulation. We have concentrated on the areas of most research activity and we apologize for nonintentionally overlooking important pieces of work.

## Establishing the Role of the VMH in Metabolic Regulation: Body Weight

The role of the VMH in appetite and body weight regulation is now firmly established. However, it took a rather long time to arrive at this point; the first indications having come from 19th century clinical observations of patients with damage to the hypothalamus (34). Hetherington and Ranson later confirmed these findings by showing that rats, with targeted electrolytic lesions, showed rapid onset of obesity (35), but only in animals with lesions confined to the ventromedial hypothalamus. The weight gain in ventromedial hypothalamus–lesioned rats was due to increased food intake and reduced sympathetic outflow.

As newer and less destructive techniques became available, the role of the VMH nucleus itself could be further investigated. SF1 knockout mice, which fail to develop a VMH, or mice with SF1 cell-specific deletion of LepR, showed an increase in body weight and adiposity (17,36). While the effect on body weight was small compared with complete loss of leptin receptors, the obesity was greatly exacerbated when the VMH LepR knockout mice were switched to a high-fat diet. The mice did not respond as wild-type littermates by reducing caloric intake or by activating diet-induced thermogenesis. This suggested that SF1 neurons are perhaps more important in adaptation to an obesogenic environment, rather than in normal body weight maintenance. Importantly, this paper was 1 of only a few to consider possible disruption to pituitary/gonadal and adrenal function, the other main tissues to express SF1 (37). This may not be relevant to all studies, but caveats regarding “extra-VMH” expression remain for metabolic studies utilizing germline modification of SF1 cells. Furthermore, as already stated, neurons in the vlVMH lose their expression of the transcription factor in adulthood (25). Thus, adult manipulations of SF1 cells in the brain will bias results towards the effects of neurons specifically in the central and dmVMH.

Perhaps the biggest issue with targeting SF1 cells, is that they form a very heterogeneous group. Thus, 1 SF1 cell type may be activated by leptin, while another may be inhibited (also see glucose sensing in later sections), so that any effect of manipulating all SF1 cells is a cumulative or compound response. It is interesting that the SF1-selective knockout of LepR (17) or cannabinoid receptor 1 (38) exacerbates the effects of high-fat diet, but the knock down of insulin receptors (39), SIRT1 nucleic acid deacetylase (40), or the FOXO1 transcription factor (41) has the opposing effect, and protects against diet-induced obesity. It is difficult to ascertain whether the compound responses to these manipulations are driven by the same neurons. The identity of specific neuronal populations involved, for example in the effect of leptin are still not fully appreciated, but there is some indication that there is an interaction with PACAP signaling (15). The expression of mRNA for PACAP in leptin-sensitive VMH neurons is inversely related to diet-induced weight gain, suggesting that PACAP^VMH^ neurons have a role in the response to leptin and an obesogenic diet. Furthermore, administration of a PACAP receptor antagonist ICV (15), or directly into the VMH (42), inhibits the acute hypophagic and hyperthermic effects of exogenous leptin.

Germline targeting of VMH neurons, which causes the life-long disruption of gene expression, can lead to compensation through redundant pathways (leading to no phenotype) or developmental effects (leading to a phenotype which is not relevant to adult function). As noted, SF1 is not expressed in the adult vlVMH (25), where other markers, notably ERα and BDNF predominate (21,43,44). VMH BDNF mRNA levels are reduced during fasting (43) and, although BDNF^VMH^ neurons do not express leptin receptors, they can be activated by leptin indirectly (45). Knocking out BDNF from SF1 neurons during the early stages of development has no impact on body weight. However, following AAV-Cre–mediated deletion of floxed *Bdnf* alleles, adult mice show a marked increase in body weight as a consequence of increased food intake (45–47). By comparison, knock down of ERα in the adult rat VMH or in embryonic mouse SF1 neurons, both cause an increase in body weight, dominated instead by a reduction in sympathetically driven thermogenesis (21,48).

## Recent Developments Regarding the VMH and Body Weight Regulation

A synopsis of this excellent body of research would suggest that the major role of the VMH may be to counteract the effects of an obesogenic diet: the normal reduction in food intake and increase in energy expenditure. The observed concomitant changes in insulin sensitivity may be dependent on body weight change, though independent effects on glucose regulation will be discussed later. In this section, we will discuss more recent studies which aim to refine the way in which VMH neurons are manipulated, and we will introduce the potential influence of thyroid hormones.

The rapid development of viral delivery, and chemogenetic and optogenetic approaches abrogates the development and redundancy issues of germline manipulation and should allow more selective manipulation. Current literature exploring the role of the VMH is still focused on SF1 neurons with very little information about other phenotypes. The dearth of other examples reflects a lack of information about additional VMH cell phenotypes, but perhaps also a number of unpublished negative results. In studies, using the viral targeting of either a stimulatory designer receptor (DREADD) or channel rhodopsin to SF1-Cre neurons, injection of the designer drug clozapine N-oxide (CNO) or low-frequency light stimulation, respectively, causes a reduction in food intake (49,50). Importantly, when SF1 neuron activity is enhanced or suppressed, using stimulatory or inhibitory designer receptors and CNO in the drinking water, effects on food intake and adiposity are seen over several weeks (50). In a series of elegant behavioral studies, the authors postulated that, in the absence of food, low SF1 neuron activity is permissive in allowing exploratory and food-seeking behavior, but that SF1 neuron activation switches behavior towards reduced exploration and food avoidance (50). We will return to the action of the VMH on different, competing behaviors in later sections, but these recent experiments do suggest that the SF1 neurons may not only be involved in counteracting the effects of an obesogenic diet.

The thyroid hormone, triiodothyronine, acts on thyroid hormone receptor α in the VMH to induce lipogenesis (via the parasympathetic nervous system) and activate brown adipose thermogenesis (via the sympathetic nervous system) (51,52). Knocking out the adenosine monophosphate (AMP)–activated protein kinase isoform, AMPKα1, in SF1 neurons has similar effects (52). However, the intracellular pathways leading to lipogenesis and thermogenesis are distinct (51). Whereas the above-mentioned effects of triiodothyronine are believed to be mediated via thyroid hormone receptor α, knocking out thyroid hormone receptor β specifically in the VMH, induces hyperphagia and lower energy expenditure due to reduced locomotion (53), suggesting that thyroid hormone can influence several aspects of energy metabolism via this nucleus.

## Establishing the Role of the VMH in Metabolic Regulation: Glucose Homeostasis

Classic studies measured changes in VMH single-unit electrical activity following intravenous glucose infusion (54,55) and were followed by an exploration of functional connections to peripheral glucose regulators. Thus, electrical stimulation of the VMH leads to both increased glucose production through increased hepatic glycogen breakdown and increased glucose uptake by peripheral tissues, such as brown adipose, heart and skeletal muscle, via sympathetic activation (56–59). There were also concomitant decreases in circulating insulin and increases in glucagon, typical of the counter-regulatory response (CRR) to hypoglycemia (60). Conversely, lesioning of the VMH resulted in increased insulin and decreased glucagon levels (61). The VMH controls these pancreatic hormones via both the parasympathetic (62) and the sympathetic pathways (61).

As we have noted already, genetic modification of VMH neurons can affect body weight and, therefore, glucose tolerance. However, key studies conducted by Borg et al. (3) demonstrated a critical role for the VMH in counter regulating hypoglycemia. The CRR is the neuro-endocrine response to hypoglycemia that involves an increase in glucagon, corticosterone, and norepinephrine levels, and a reduction in insulin secretion. Chemical lesions of the VMH suppressed the endocrine CRR following hypoglycemia in rats (3), whereas CRRs were initiated by the induction of the glucopenia through delivery of a nonmetabolizable form of glucose (2-deoxy glucose) directly into the VMH (63). Most importantly, local perfusion of glucose into the VMH prevented CRR following insulin-induced hypoglycemia (64). Since most neurons in the VMH are glutamatergic (8), knocking out the glutamate transporter, VGLUT, selectively in SF1 neurons, resulted in an impaired CRR to insulin–induced hypoglycemia (65).

Early investigations into profiling the neurocircuits that regulate glucose, identified neurons which are intrinsically sensitive to changes in glucose (54,55,66). Glucose-sensing neurons are broadly classified as being either “glucose excited (GE)” or “glucose inhibited (GI).” The former increase their activity in response to rising glucose (hyperglycemia), whereas the latter are inhibited. Conversely, in the presence of low glucose (hypoglycemia), GI neurons are excited and GE neurons are inhibited. As glucose levels in the brain are maintained at approximately 30% of systemic levels (67,68), these neurons are responsive to glucose levels in the range of 0.1 mM to 5 mM (69,70). Within the VMH, there are additional subsets of glucose-responsive neurons which are not intrinsically glucose sensing. Thus, 1 subset is presynaptically excited in response to decreased extracellular glucose levels (PED), and 2 further subsets are either presynaptically excited or inhibited, respectively, in response to increased extracellular glucose (71). In rats, 14% of VMH neurons are GE, 3% are GI, 14% are PED, and another 18% are also synaptically driven to respond (71). A more recent, multi-electrode array study made extracellular recordings from mouse slices in vitro (72). Overall, 15% to 60% were GE or presynaptically excited by increasing glucose, whereas only 2% to 7% were GI or PED. A rise in systemic glucose concentrations causes an increase in Fos expression (mainly in the dmVMH), whereas hypoglycemia causes a significant reduction in neuronal activation (73,74). Interestingly, no difference in the spatial distribution of GI neurons was seen in the multi-electrode recordings (72).

## Recent Developments Regarding the VMH and Glucose Regulation

Currently, it is not possible to allocate definitive glucoregulatory roles to the different classes of glucose-sensing and glucose-responsive neurons of the VMH, and it remains possible that the classes are themselves phenotypically diverse. It is likely that there is some overlap between GE and leptin-sensitive neurons in the dmVMH (73). The intrinsic glucose- and leptin-sensing ability of VMH neurons requires K_ATP_ channels and phosphatidylinositol-3-kinase (75). Furthermore, Toda et al. (73) describe the importance of uncoupling protein 2-dependent mitochondrial fission and reduced reactive oxygen species in these VMH neurons in mediating the downstream increases in glucose uptake by peripheral tissues. By comparison, GI neurons seem more involved with the increase in hepatic glucose production via hypoglycemia-associated CRR (76). Their intrinsic glucose-sensing ability requires AMP-activated protein kinase, which links with nitric oxide–guanylate cyclase signaling to cause depolarization through the closure of a membrane chloride channel (16).

The same caution, as before, is required in interpreting recent studies which manipulate SF1 neurons, due to the heterogeneity of VMH cell types. That said, Meek et al. demonstrated, in a very elegant study, that the optogenetic inhibition of adult SF1 neurons has no effect on fasting blood glucose levels, but impairs the ability of mice to recover from a modest insulin-induced hypoglycemia (77). These data would support the notion that a major role of the VMH is to protect against hypoglycemia. Meek et al. also optogenetically stimulated SF1 cell bodies, or selectively activated SF1 terminals in the anterior bed nucleus of the stria terminalis (aBNST), and saw large increases in circulating glucose, concomitant with secretion of glucagon and corticosterone (2 counter-regulatory hormones) (77). Two more papers have confirmed that photostimulation of SF1 neurons causes hyperglycemia, while the stimulation of VMH cell bodies containing NOS1 (78) or cholecystokinin receptor B (CCK_B_) (11) causes even greater increases in glucose. However, it should be noted that in each of these papers very high stimulation parameters (40 Hz for 1 hour) were utilized, which will drive neurons at a supraphysiological pace. Optogenetic stimulation of SF1 neurons at frequencies greater than 5 Hz will override intrinsic firing patterns, to drive a state of aversion, autonomic activation and powerful escape behavior (50,79).

The use of chemogenetics does not provide the same possibility for temporal regulation but, in the absence of patterned optical stimulation, will provide firing patterns more reflective of endogenous activity. Thus, it noteworthy that no papers describe an increase in glucose following chemogenetic activation of SF1 neurons. In fact, Coutinho and colleagues found activation of SF1 neurons with a stimulatory DREADD caused decreases in glucose (49), which contradicts the optogenetic studies. They concluded that chemogenetic stimulation of SF1 neurons increases insulin-dependent uptake of glucose by peripheral tissues. This concurs with Toda et al., who had shown that uncoupling protein 2 expression in SF1 neurons is critical for the ability of the body to maintain insulin sensitivity. These researchers found that inhibition of SF1 neurons with an inhibitory DREADD increases glucose in a glucose-tolerance test (73), though other groups have reported no effect on basal glucose levels (77,80).

PACAP^VMH^ neurons are GI and, although activating them with a stimulatory DREADD inhibits insulin (which is the first CRR to falling glucose levels), this manipulation does not release glucagon (a CRR to a deeper hypoglycemia) or affect basal glucose levels (16). Instead, activating PACAP^VMH^ neurons does increase glucose during a glucose tolerance test. These PACAP^VMH^ neurons are also activated by cholecystokinin (16). As mentioned, supraphysiological (40 Hz) photostimulation of CCK_B_^VMH^ neurons causes hyperglycemia, whereas PACAP^VMH^ cells only increase their firing approximately from 1 to 3 Hz, in response to a physiological drop in glucose (16). More importantly, perhaps, disabling CCK_B_^VMH^ neurons with Cre-dependent tetanus toxin suppresses hepatic glucose production and the CRR to insulin-induced hypoglycemia (11). VMH GI neurons are dependent on nitric oxide signaling to show inhibition in response to rising glucose, though there are far more NOS1 positive than GI neurons in the VMH (81,82). Stimulating NOS1^VMH^ neurons optogenetically elicits hyperglycemia; however, this is accompanied with freezing behavior (78). This was investigated more closely by selectively stimulating projection fields of NOS1^VMH^ neurons, in the aBNST or the periaqueductal gray (PAG) in the midbrain. A 40-Hz stimulation of fibers innervating the aBNST elicited powerful hyperglycemia, but no freezing behavior. Whereas, projection-specific stimulation of NOS1^VMH^ fibers in the PAG showed even more extreme hyperglycemia and freezing behavior. A 2-Hz photostimulation of SF1 neurons decreases feeding and induces place preference, but was not reported to affect glucose (50). Stepped increases in stimulation above 4 Hz causes anxiety, place avoidance, freezing and escape behaviors (50,79). Finally, He and colleagues (83) have concentrated on VMH neurons which express ERα. These neurons are concentrated in, but not exclusive to the vlVMH, and appear not to express SF1 during adulthood (25). Interestingly, all ERα cells in the vlVMH are intrinsically glucose sensing, with 57% GI and 43% GE. Each is sufficient to drive changes in circulating glucose via their connections with the hypothalamic arcuate nucleus and the midbrain dorsal raphe nucleus, respectively. This sets them out as being distinct from PACAP^VMH^ or NOS1^VMH^ cells which project to alternative brain regions (16,78).

While this large body of work leaves no doubt that the VMH, especially GI neurons, can drive CRR to hypoglycemia, its role as the primary sensory limb of this function has been challenged. It has been suggested that a glucose regulatory effector system, comprising glucose sensing neurons located in the arcuate nucleus, median eminence and nucleus of the tractus solitarius, in co-operation with nerve cells innervating the hepatic portal system, are crucial to the CRR response (84). Such peripheral sensors are able to detect changes in circulating glucose and accordingly modify CRR via the central glucoregulatory neurons located in the VMH and other brain regions (85). Moreover, the glucose-sensing ability of VMH neurons is dispensable and may only become important in pathological conditions when the peripheral system fails to rescue falling glucose levels. Alternatively, GI and GE neurons could measure central glucose levels for benchmarking against peripheral levels and to modulate the CRR response accordingly (84).

## Establishing the Role of VMH in Social Behavior: Aggression

Aggressive behavior is preserved across species and is implicated in self-preservation, for example, through adapting food seeking and territorial dominance. Understanding how and what causes the brain to engage such behavior could help develop treatments for ailments in which aggression is exacerbated and can pose a threat to life.

Attack behavior was observed when the vlVMH of rats was electrically stimulated (86). The “resident-intruder” test in mice, showed a strong induction of c-*fos*, particularly in the vlVMH and an increase in electrical spiking activity when a male intruder is introduced into the cage (7). As the encounter progressed to an attack, the neurons exhibited a further increase in spiking activity (7,87). Unlike the progressive increase in spiking activity observed in response to a male intruder, introduction of female intruder resulted in a decrease in spiking activity in these neurons as the interaction progressed towards mating (7). Selective optogenetic stimulation of vlVMH neurons caused in an increase in attack response not only towards males (as seen during spontaneous attacks), but also towards castrated males, females and inanimate objects. Whereas, silencing vlVMH neurons caused a reduction in aggressive attack behavior (7).

## Recent Developments Regarding the VMH and Aggressive Behavior

As in the case of metabolic regulation, in order to decipher the underlying neuronal circuitry that controls aggressive behavior, it is imperative to identify the different neuronal populations and pathways involved. Taking advantage of the fact that neurons which express both ERα and progesterone receptor (PR) are limited mostly to the vlVMH, several studies have investigated the role of these neurons in VMH-controlled aggression. Rodents with chemogenetic or optogenetic inhibition of ERα (88,89) or PR (6) neurons in the VMH show a reduction in aggressive attack behavior. Whereas, activation of these neuronal populations increases attacks and decreases the latency to attack (88,90).

Independent from attacking behavior, other forms of aggressive behavior, such as defensive rage, also are modulated by the VMH. High-frequency optical stimulations (20 Hz) of SF1 neurons in the in the dorsomedial and central division of the VMH induces defensive/avoidance behaviors, such as running around the cage perimeter, jumping, and escaping (50,79,91). It is noteworthy to indicate that these behaviors are observed only at high-frequency stimulation. Instead, with optical stimulation at a lower frequency (5 Hz), mice exhibit freezing behavior (79). Similar frequency-dependent effects are observed in females when ERα ^vlVMH^ neurons are stimulated: high-frequency stimulation increases the propensity of attacking rather than mating (88,92). These observations suggest that the VMH could possibly use frequency-dependent mechanisms to modulate different aspects of social behavior. However, the paper by Wang et al. describes beautifully a cautionary note regarding how high-frequency stimulation can lead to back propagation of action potentials, which can affect VMH collaterals differentially (79). Another recent study in mice has investigated the role of PACAP neurons in driving an intra-VMH circuit involved in aggressive behavior (93). It is suggested that PACAP neurons in the central part of the VMH, projecting to the vlVMH, form an integral pathway involved in the circadian regulation of aggressive behavior. Unlike the observations made using SF1-Cre mice (79), chemogenetic stimulation of PACAP-Cre neurons in the central VMH, induces an increase in total attack time during the resident-intruder test. This refinement once again highlights the fact that inferences drawn from manipulations of SF1 neurons may not provide enough detail to delineate complex neurocircuits.

Another important factor is to understand how the neurons within this area orchestrate the various sensory cues and motor responses, for example during the investigation and action stages of aggression. The vlVMH receives inputs from both the main and the accessory olfactory systems (94), allowing it to process vital information regarding the investigative phase. This is crucial since, as there is a strong overlap between the mating and aggression circuits, it allows the VMH to process sensory input to differentiate between threatening and social cues. For example, as mentioned earlier, it is observed that while activity in vlVMH neurons increases when investigating a resident male, the activity decreases when a female is presented into the cage (87). Recent work has explored a possible pathway that drives motor responses specifically during an attack. Glutamatergic, VGlut2^vlVMH^ neurons, which project to the lateral PAG, drive the biting response during attack (95). Unlike the PAG neurons, which are active only during the attack phase, the vlVMH neurons show increased activity during both social interactions and active attacks. Once again, such findings emphasize the importance of identifying different neuronal populations within the VMH to understand how the nucleus integrates various social cues to instigate the required response.

## Establishing the Role of VMH in Social Behavior: Sexual

Early lesion studies showed that the VMH is involved in the modulation of sexual behavior in female rats (96). The VMH was observed to be crucial in modulating the female lordosis reflex which is required for successful mating (97,98). Furthermore, in female rats, electrical stimulation of the VMH facilitated lordosis (99), while sexual behavior induced Fos expression (100,101). In males, the VMH was observed to be vital for sociosexual behaviors like scent marking and partner preferences, but with limited effect on copulatory behaviors, such as mounting (102,103). As a result, early studies on the role of the VMH in driving sexual behavior focused on females.

Knocking out SF1 neurons affected both fertility and reproductive behavior in female mice only (104), blocking the action of estrogen and progesterone in driving sexual behavior (105,106). Further studies implicated different pathways with distinguishing characteristics: ERα-expressing neurons projecting to the PAG (107), non-ERα cells, and unidentified neuronal populations which do or do not project to the PAG (100, 108). Various other phenotypic markers have been identified in the context of lordosis behavior. These include oxytocin receptor–expressing neurons (109), and NOS1 neurons (110), both of which promote female sexual behavior.

## Recent Developments Regarding the VMH and Sexual Behavior

Due to the clear overlap in VMH neurocircuitry involved in driving aggressive and sexual behaviors (7), more recent studies have tended to compare mating behavior in both males and females. Optogenetic stimulation of ERα ^vlVMH^ neurons in male mice increases sociosexual and mounting behavior (88). However, this behavior is observed only at low-intensity stimulations, as higher intensity stimulation results in increased aggressive behavior (88). In female mice, optogenetic stimulation of ERα ^vlVMH^ neurons also increases sexual behavior (88,92). Furthermore, by using fiber photometry, increased activity is observed in both ERα ^vlVMH^ and PR^vlVMH^ neurons during female sexual behavior (92,111). Anterograde and retrograde labelling shows dense projections of these neurons to the anteroventral periventricular nucleus (6,92), another brain region anterior to the hypothalamus that is crucial for modulating female sexual behavior.

## The VMH as A Driver of Sexual Dimorphism in Homeostatic Signals

The fact that the VMH integrates sex differences into many of its functions adds to its complexity as a master regulator. For example, the VMH is often investigated with regards to specific types of male behavior, such as territorial aggression. The relative lack of research looking at the neurocircuitry engaged by females may be a consequence of the fact that aggression in humans is more commonly attributed to males. Nevertheless, an appreciation of gender differences is vital for understanding other types of conflict, such as maternal aggression. Recently, Lin et al. have carried out excellent studies of sexual dichotomy in aggressive behavior (92). Using a modified resident-intruder test, they find that activating ERα ^vlVMH^ neurons is both necessary and sufficient to induce aggressive behaviors in female Swiss Webster mice. If the neurons are activated in in females on a C57Black6 background, they display sexual rather than attacking behaviors. Furthermore, they observe that unlike in male mice where there is an overlap between neurons in the vlVMH involved in mating and aggressive behavior (7,87,90), in female mice there is a clear anatomical and molecular distinction between neurons driving these 2 different types of behavior (92).

The high density of estrogen receptor in the VMH, not only drives sex-specific behaviors, but allows the modulation of metabolic homeostasis by estrogen. Estradiol injections cause a reduction in body weight, but this is attenuated in rats with VMH lesions (112). Likewise, ovariectomized mice put on weight, and this is enhanced by selective chemogenetic silencing of VMH ERα receptors (21,113). Knocking out ERα selectively in SF1 neurons shows similar effects, with an increase in adiposity only in female mice (48). The single-cell RNA sequencing of SF1-Cre cells, identified a neuronal cluster expressing *Rprm,* which colocalizes with ERα, in the vlVMH only of female mice (26). When *Rprm* is knocked down, an increase in body temperature (with no change in activity) is observed in female, but not male mice (26). Two other sexually dimorphic neuronal clusters were observed in this study: those containing *Tac1* or *Pdyn* (tachykinin and prodynorphin precursor genes, respectively). Chemogenetic activation of Tac1^vlVMH^ neurons increases locomotor activity, but with no changes in brown adipose-dependent thermogenesis. Whereas, knocking down *Tac1* in the VMH reduces locomotor activity only in females (114). Further analysis of these different neuronal clusters hopefully will identify other such gender-specific differences in metabolic regulation.

The effect of sex hormones on the VMH further extends to glucose homeostasis and the hypoglycemia CRR. Selectively limiting ERα activity in the VMH results in an increase in circulating glucose levels (21,115). Furthermore, electrophysiological recordings in the VMH have shown a sexual dimorphism in the sensitivity of GI and not GE neurons to detect hypoglycemia, with male mouse neurons generally increasing their excitability at lower glucose concentrations compared with female mice (116).

## Conclusion

It is well established that destroying the VMH reduces aggression/fear and increases body weight. However, such manipulations do not reveal whether the VMH has a homeostatic function to regulate metabolism, or to drive competing behaviors. More refined, loss-of-function experiments, that utilize the chemogenetic inhibition or ablation of SF1 neurons selectively, do induce a reduction in fear behavior and an increase in food intake. Conversely, high-frequency photostimulation of SF1 neurons reduces food intake, but can produce anxiety, defensive escape behaviors, aggression, and increased blood glucose—all symptomatic of heightened arousal, sympathetic activation, and the “fight or flight” response.

Switches in behavior are dependent on the energy status of the animal, meaning the SF1 neurons must sample the *internal milieu* before determining behavioral output. Thus, it is tempting to speculate that other circuits drive appetite, while activation of the VMH pushes the animal towards alternative, competing behaviors, so long as a critical energy levels are maintained. A dangerous threat is likely to drive either aggression or escape. However, competing behaviors may not only be in response to threats. An opportunity to reproduce may activate VMH pathways that favor sexual behavior. An interesting peculiarity is the threat of hypoglycemia, which causes a VMH-mediated endocrine response while still allowing other circuits to induce feeding.

**Figure 2.**
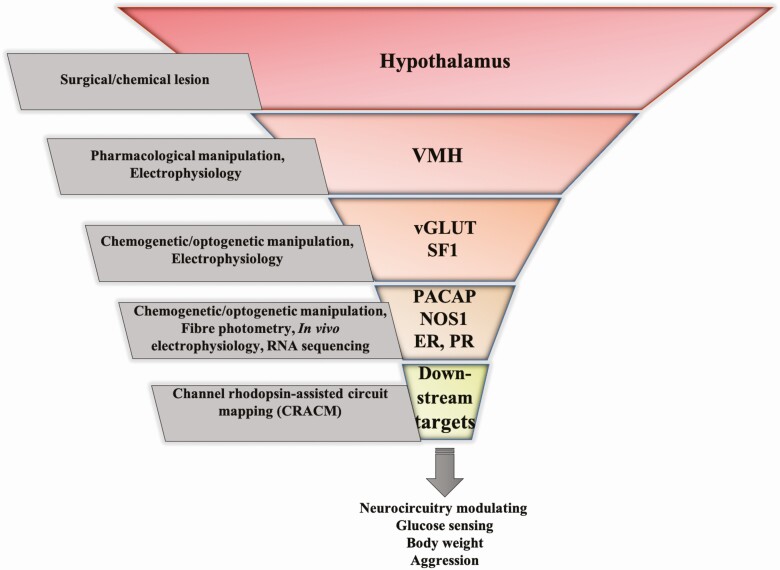
The range of techniques used over time that have helped identify the role of VMH neurons in maintaining glucose, body weight and innate behavioral responses. As newer techniques evolved, we have been able to investigate the VMH in greater detail.

As discussed, manipulation of SF1 neurons will modulate most, if not all, of the above-mentioned functions simultaneously. Downstream circuits may produce opposing effects and/or competing behaviors (see sexual vs aggressive behavior). The same is clearly true for manipulation of other phenotypically labelled neurons (including PACAP, NOS1, and ERα), which are probably themselves very mixed populations of cells, some of which overlap. While these markers are a starting point for unravelling this complex nucleus, further depth in the phenotypic profiling of the VMH is vital. Progress is also achieved by looking at specific neuron projections. VMH neurons project to multiple other brain sites, apparently with much collateralization. Optogenetic stimulation of fibers for circuit mapping can dissect apart some of these pathways, but care must be taken in interpreting the responses to high-intensity stimulation. While there is a suggestion that VMH neuronal pathways may exhibit frequency-dependent functioning, supraphysiological activational frequencies can lead to “back propagation” of action potentials and drive multiple collaterals (79). Careful titration of stimulus parameters, preferably within the physiological range, should be attempted if possible. Figure 2 suggests an ever increasing refinement of our knowledge as technologies have evolved. Let us hope that new developments will carry us closer to a complete understanding of this fascinating brain structure.

## Data Availability

Data sharing is not applicable to this article as no datasets were generated or analyzed during the current study.

## Abbreviations

AAV: adeno-associated virus
aBNST: anterior bed nucleus of the stria terminalis
AMP: adenosine monophosphate;
BDNF: brain-derived neurotrophic factor
CCK: cholecystokinin
ChR2: channel rhodopsin
CNO: clozapine N-oxide
CRR: counter-regulatory response(s)
DREADD: designer receptors exclusively activated by designer drugs
ER: estrogen receptor
GE/GI: glucose excited/inhibited, intrinsically sensitive neurons
LepR: leptin receptor
NOS1: nitric oxide synthase 1
PACAP: pituitary adenylate cyclase–activating peptide
PAG: periaqueductal gray
PED: presynaptically excited
PR: progesterone receptor
SF1: steroidogenic factor-1
TR: thyroid hormone receptor
UTR: untranslated region
VGLUT: vesicular glutamate transporter
VMH: ventromedial nucleus of the hypothalamus
vlVMH/dmVMH: ventrolateral/dorsomedial portion of the VMH

## Additional Information

***Disclosures:*** The authors have nothing to disclose.
